# *Houttuynia cordata* Extract Alleviates *SVCV* Infection by Modulating the *RARα-A*/Type I Interferon Axis in Common Carp

**DOI:** 10.3390/ani16152350

**Published:** 2026-08-01

**Authors:** Chi Zhang, Yaqian Bai, Qian Liu, Yanwei Zhang, Xianlin He, Ya Zhou, Xianqin Hu, Jiaqi Zhang

**Affiliations:** 1 Hubei Key Laboratory of Animal Nutrition and Feed Science, School of Animal Science and Nutritional Engineering, Wuhan Polytechnic University, Wuhan 430023, China; zhch@whpu.edu.cn (C.Z.); baiyqq@126.com (Y.B.); 19835053425@163.com (Q.L.); 2 College of Fisheries, Xinyang Agriculture and Forestry University, Xinyang 464000, China; zhangyw03@163.com; 3 College of Animal Science and Technology, Chongqing Three Gorges Vocational College, Chongqing 404155, China; hexianlin@cqsxzy.edu.cn (X.H.); asian_chou@outlook.com (Y.Z.)

**Keywords:** *Houttuynia cordata*, spring viremia of carp virus (*SVCV*), *Common carp*, immunity

## Abstract

*Spring viremia of carp virus* (*SVCV*) poses a major threat to aquaculture industry, triggering severe economic losses and substantial damage to fish health. Effective antiviral therapeutic approaches are currently insufficient in aquaculture, highlighting an urgent demand for viable intervention strategies. This study assessed the antiviral and immunomodulatory potential of *Houttuynia cordata* against *SVCV* in common carp. Combined cellular and in vivo fish experiments demonstrated that *Houttuynia cordata* markedly suppresses *SVCV* replication, reduces viral titer, and enhances disease resistance of carp. The extract appears to suppress *RARα-A*, upregulate *IFN-1*/*CXCL11*, and recruit T lymphocytes via *CXCL11*, thereby enhancing innate and adaptive immunity against *SVCV*. Experimental results indicate that *Houttuynia cordata* alleviates hemorrhagic symptoms induced by *SVCV* infection. Dietary supplementation with this herb realizes dual functions of viral suppression and immune regulation for carp aquaculture. The findings provide a practical prevention and control strategy against *SVCV* infection and offer novel insights for research on green antiviral agents. Furthermore, this study verifies the potential value of *Houttuynia cordata* in improving biological safety and facilitating sustainable development of aquaculture, laying a solid foundation for the development of eco-friendly antiviral preparations.

## Simple Summary

Viral diseases frequently break out in carp farming, severely damaging fish health and causing huge economic losses to aquaculture producers. Relying on chemical drugs for disease prevention easily causes environmental pollution and drug residue problems, so safe and natural immune-enhancing additives are urgently needed. This study explored the protective effect and working principle of a common herbal plant extract on virus-infected carp. The results showed that this herbal extract can activate the fish’s internal immune regulation process, effectively inhibit virus proliferation and reduce viral damage to fish tissues. It can block virus growth by interfering with viral genetic material and protein production. This work reveals the clear immune regulation mechanism of the herbal extract, provides a new safe green prevention and control method for carp viral diseases, helps reduce chemical drug use, and promotes the healthy and sustainable development of ecological aquaculture.

## 1. Introduction

Common carp (*Cyprinus carpio*) is an economically significant freshwater fish species cultured extensively worldwide. However, the rapid expansion and intensification of carp aquaculture have increasingly exposed farmed populations to diverse viral pathogens. Among these, spring viremia of carp virus (*SVCV*) has emerged as a major threat, causing substantial economic losses to the cyprinid farming industry. *SVCV* belongs to fish rhabdovirus with a genome length of approximately 11 kb [1]. *SVCV* typically infects cyprinid fish at water temperatures of 10–15 °C in spring, with young fish being the most susceptible [2]. Cyprinid fish infected with the virus display clinical signs including body surface hemorrhage, exophthalmos, abdominal distension and gill congestion [3].

Antibiotics, chemical drugs and vaccination are the main measures for fish disease prevention and control under intensive aquaculture mode [4]. These approaches are prone to drawbacks including drug residues, drug resistance and high costs. Hence, it is urgent to explore green, efficient and economical strategies to promote eco-friendly and healthy aquaculture. Chinese herbal medicines feature low toxicity, negligible drug resistance and low cost, and exert no adverse impacts on aquaculture water and human health. Existing studies have demonstrated that Chinese herbal medicines possess remarkable application potential against *SVCV*. For instance, saikosaponin D can alleviate cellular morphological damage and reactive oxygen species production induced by *SVCV*, inhibit viral replication and improve survival rate in infected zebrafish and common carp [5]. Schisandrin A can dualistically inhibit *SVCV* replication and improve the survival rate of juvenile carp by enhancing innate immunity, exerting antioxidant effects and protecting mitochondrial function [6]. Nevertheless, few studies have reported the antiviral effects of *Houttuynia cordata* against *SVCV* to date.

*Houttuynia cordata*, a traditional Chinese herbal medicine, possesses extensive pharmacological activities including antibacterial, antiviral, anti-inflammatory, and immunomodulatory effects [7], with particularly prominent antiviral properties [8]. Studies have shown that *H. cordata* extracts can inhibit Salmonella invasion by regulating virulence genes, repair intestinal damage and alleviate inflammation, which can be applied to green prevention and control of avian salmonellosis [9]. Appropriate supplementation of *H. cordata* powder in diet can markedly enhance cutaneous mucosal and serum immunity of Nile tilapia [10]. Furthermore, crude extracts of this herb exert remarkable inhibitory effects against both HSV-1 and HSV-2, while Houttuynoid A, a flavonoid isolated from *H. cordata* Thunb, exhibits potent activity specifically against HSV-1 [11]. Given the immune-enhancing effects of *H. cordata* on fish organisms, this study investigated the intervention effects and underlying mechanisms of *H. cordata* extract against *SVCV* infection through transcriptomic analysis, combined with the evaluation of immune indices, viral load and host disease resistance responses. The findings aim to provide novel insights into the green prevention and control of *SVCV*-associated diseases in intensive carp aquaculture, as well as a theoretical basis for the application of *H. cordata* in the prevention and treatment of fish diseases.

## 2. Materials and Methods

### 2.1. H. cordata Extract

The *H. cordata* extract was jointly extracted and prepared by our laboratory and Chongqing Three Gorges Vocational College. Briefly, 2000 g fresh *H. cordata* was steam-distilled, with 2000 mL primary distillate collected and redistilled to obtain ~1000 mL redistillate. After adding 7 g sodium chloride and 2.5 g polysorbate 80, the solution was diluted to 1000 mL with water for injection, filtered, sealed in ampoules and sterilized to make a colorless clear aqueous extract, which was stored sealed, protected from light, in a cool place. Its GC fingerprint had four characteristic peaks (relative retention times: peak 1 = 0.890, peak 2 = 0.927, reference peak S (methylnonanone) = 1.000, peak 3 = 1.029), with consistent GC profiles and physicochemical properties across batches. The main active components are volatile oils (houttuynin, methylnonanone), flavonoids, phenolic acids and polysaccharides.

### 2.2. Cells and Viruses

Epithelioma papulosum cyprini (*EPC*) cells were preserved long-term in liquid nitrogen in our laboratory. *EPC* cells were cultured in complete M199 medium (Gibco, Grand Island, NY, USA) supplemented with 10% fetal bovine serum (FBS, Yeasen Biotechnology Co., Ltd., Shanghai, China), 100 U/mL penicillin, and 100 μg/mL streptomycin (Gibco, Grand Island, NY, USA), and maintained at 25 °C for subculture. Experimental common carp were purchased from a local market. The prototype *SVCV* strain ATCC VR-1390 was cultured in *EPC* cell monolayers at 28 °C; virus harvest was performed once cytopathic lesions induced by the virus covered more than 80% of the cell monolayer. The viruses were then harvested and subjected to 3 freeze–thaw cycles. Following the collection of the cell culture medium, the samples were centrifuged at 10,000 g for 10 min at 4 °C. The resulting supernatant was extracted and stored at −80 °C (MOI = 0.1).

### 2.3. Primers and Antibodies

All *RT-qPCR* primers involved in this study (Table 1) were self-designed by the authors. The complete CDS sequences of target genes were retrieved from the NCBI GenBank database. Primer 5 software was used for primer design with standard qPCR parameters: amplicon length 80–200 bp, primer length 18–22 nt, and annealing temperature controlled between 58 and 62 °C. After preliminary screening in Primer 5, the specificity of each primer pair was further verified via NCBI Primer-BLAST (https://www.ncbi.nlm.nih.gov/tools/primer-blast/, accessed 08 July 2025.) to avoid non-specific amplification. Finally, all lyophilized primer powders were synthesized using Tsingke Biotechnology Co., Ltd., Wuhan, China. The primary antibodies against *SVCV*-N and *SVCV*-G proteins were mouse monoclonal antibodies provided by Huazhong Agricultural University and preserved in our laboratory. β-actin antibody and HRP-conjugated secondary antibody were obtained from Proteintech Group, Wuhan, China.

### 2.4. Cell and Virus Infection

#### 2.4.1. Cellular Prophylaxis

*EPC* cells were routinely cultured and seeded into 12-well plates. The experimental group was treated with 1 μL of 0.2 g/L *H. cordata* extract, whereas the control group was administered an equal volume of blank solvent devoid of the extract to eliminate any experimental interference caused by the solvent itself. Cells were incubated at 26 °C. After forming a confluent monolayer, cells were infected with 0.5 μL *SVCV* (MOI = 0.1) at a titer of 10^6^ TCID_50_/mL. Cell samples were collected and labeled 24 h post-infection for subsequent assays.

#### 2.4.2. Infection of *EPC* Cells with SVCV for 3 h, 12 h and 24 h

*EPC* cells were routinely subcultured in 12-well plates. After reaching full monolayer confluence, 1 μL of 0.2 g/L *H. cordata* extract was supplemented into the experimental group, whereas the control group was administered an equal volume of blank solvent devoid of the extract to eliminate any experimental interference caused by the solvent itself. Cells were incubated at 26 °C. After 12 h, cells were infected with 0.5 μL *SVCV* (MOI = 0.1) with a titer of 10^6^ TCID_50_/mL. Cell samples were collected and labeled at 3 h, 12 h and 24 h post-infection for subsequent experiments.

#### 2.4.3. Antiviral Effect of *H. cordata* Extract with Different Concentrations on EPC Cells

*EPC* cells were routinely subcultured into 12-well plates and incubated at 26 °C for 12 h until forming a confluent monolayer, then the culture medium was discarded. The control group was supplemented with 400 μL medium and 0.5 μL *SVCV* (MOI = 0.1) at a titer of 10^6^ TCID_50_/mL. The experimental groups were treated with 400 μL *H. cordata* extract at concentrations of 0.2 g/L; 0.2 × 10^−3^ g/L and 0.2 × 10^−6^ g/L combined with 0.5 μL *SVCV*. Cells were harvested and labeled after 12 h incubation for subsequent tests.

#### 2.4.4. Viral Challenge Experiment of Common Carp

Three experimental groups were designed in this study: virus-only preliminary challenge group (P), 3% *H. cordata* supplemented diet treatment group (T), and basal diet untreated infected control group (C). Fifteen carps with uniform body weight and size were randomly allocated to each group using a random number table to avoid grouping selection bias. For the preparation of herbal feed for Group T, 3% *H. cordata* extract was evenly sprayed onto basal commercial carp feed, followed by thorough stirring for homogeneous mixing. The medicated feed was air-dried at room temperature in a dark and ventilated space to prevent degradation of active ingredients and stored at 4 °C for short-term preservation. Fish in Group T and Group C received twice-daily feeding with their respective diets over a 3-week consecutive dietary pretreatment period prior to *SVCV* challenge. In contrast, Group P underwent no dietary pretreatment and was directly injected with *SVCV* (MOI = 0.1). Obvious pathological lesions were observed in carp of Group P at 4 days post-challenge (Figure A2), and cumulative mortality began to appear at 7 days post-challenge.

All experimental fish were reared in independent culture tanks, with each tank defined as one biological replicate. The water temperature was stably maintained at 22 ± 0.5 °C. Continuous aeration kept the dissolved oxygen concentration above 6.0 mg/L, and a low stocking density was adopted to alleviate stress responses of fish. Fish were fed twice daily with a daily ration accounting for 2% of their body weight. *SVCV* (MOI = 0.1) inoculation was performed via intraperitoneal injection, and an equivalent viral dosage was adopted across all virus-challenged groups.

After *SVCV* injection, fish mortality was recorded twice daily in the morning and evening. No mortality was observed in either Group T or Group C throughout the experiment. Moribund fish were humanely euthanized by overdose of MS-222 anesthetic. Tissue sampling was implemented at 4 and 7 days post-challenge. The sampling procedure was as follows: each fish was photographed to record external macroscopic lesions, followed by dissection to collect tissue samples including gill, liver, spleen, head kidney, muscle and intestine. Random pathogen screening was conducted before the formal trial, confirming that all experimental carps were free of *SVCV* and other prevalent aquatic pathogenic bacteria.

This study was reviewed and approved by the Animal Ethics Committee of Wuhan Polytechnology University. All experimental procedures complied with the requirements of animal welfare and ethics, ethical approval number: WPU202507006.

### 2.5. Plasmid Transfection

*Escherichia coli* harboring *RARα-A* plasmid was cultured and amplified, and plasmid DNA was subsequently extracted for transfection. Solutions A and B were prepared with Opti-MEM medium, *RARα-A* plasmid DNA and PEI reagent following the protocols provided by Tsingke Biotechnology Co., Ltd., Wuhan, China. Solution B was thoroughly mixed with Solution A and incubated at room temperature for 20 min to form DNA–PEI complexes. The prepared complexes were added into 6-well plates and incubated for 6 h. After transfection, the culture medium was replaced with fresh complete medium, and cells were continuously cultured for another 24 h. Empty vector was transfected into *EPC* cells as the control group. Western blot assay was used to detect the expression level of *RARα-A*. After successful overexpression of the target gene, cells were infected with *SVCV*. Cell samples were collected to determine viral load as well as the expression profiles of immune and inflammatory related genes, so as to elucidate the potential regulatory mechanism of *RARα-A* underlying the anti-*SVCV* effect mediated by *H. cordata* extract.

### 2.6. RARα-A Gene siRNA Knockdown Assay

To explore the function of *RARα-A* in the regulation of anti-*SVCV* response mediated by *H. cordata* extract in common carp, transient siRNA transfection was applied to knock down *RARα-A* expression in *EPC* cells. *EPC* cells at logarithmic growth phase were rinsed with PBS, digested by trypsin, centrifuged and resuspended. Cells were seeded evenly and cultured at 25 °C until cell confluence reached 70–80% before transfection. Specific siRNA targeting *RARα-A* and negative control *siRNA* (NC-siRNA) were designed and synthesized by Tsingke Biotechnology Co., Ltd., Wuhan, China. The sense sequence of *RARα-A* siRNA-1 was 5′-GCAAAUACACCACGAGUAA-3′, and the antisense sequence was 5′-UUACUCGUGGUGUAUUUGC-3′. The sense sequence of *RARα-A* siRNA-2 was 5′-GCUCAGUGCUAUAUGCCUA-3′, and the antisense sequence was 5′-UAGGCAUAUAGCACUGAGC-3′. The sense sequence of negative control siRNA was 5′-UCUCCGAACGUGUCACG-3′, and the antisense sequence was 5′-AGGUGACACGUUCGGAATT-3′. According to the reagent instructions, siRNA at the concentration of 200 nmol/L was transfected into *EPC* cells using PEI. The knockdown efficiency was verified by *RT-qPCR* through detecting *RARα-A* mRNA level at 24 h post transfection. After effective gene knockdown, cells were challenged with *SVCV*. Cell samples were collected to detect viral load and expression of immune and inflammatory related genes, so as to clarify the potential regulatory mechanism of *RARα-A* in the anti-*SVCV* effect induced by *H. cordata* extract.

### 2.7. Virus Titer Determination

Culture supernatants of tested samples were collected and stored at −80 °C for later use. A total of 1 × 10^4^ cells were seeded into each well of 96-well plates with a volume of 100 μL per well. After cell monolayer formation, virus inoculation was performed. The frozen viral solution was serially diluted 10-fold with serum-free M199 medium to obtain gradients ranging from 10^−1^ to 10^−10^. Cells were rinsed twice with M199 medium, then 100 μL diluted viral solution was added into each well, and blank control was set using blank dilution medium. Cytopathic changes were observed every 12 h for consecutive 7 days, and lesion-positive wells were recorded. The viral titer was calculated based on the Kärber formula.

### 2.8. Transcriptomic Analysis

Cell samples were harvested at 24 h post viral challenge. Total cellular RNA was extracted via Trizol method. Total eight samples consisting of four biological replicates in the *H. cordata*-treated group (H_24 h *EPC*) and four biological replicates in the untreated control group (N_24 h *EPC*) were prepared from *EPC* cells and subjected to paired-end RNA sequencing with a read length of 2 × 150 bp on the Illumina NovaSeq platform (Illumina Inc., San Diego, CA, USA). Raw reads ranging from 40,038,438 to 43,451,684 per sample were quality-controlled with Cutadapt v4.6 to remove adapters and low-quality reads with average base quality below Q20 or length shorter than 50 bp, yielding clean reads accounting for over 99.10% of raw data with Q20 > 99.19% and Q30 > 96.53% across all samples. The raw RNA-seq sequencing data generated in this study have been deposited in the NCBI Sequence Read Archive (SRA) database under BioProject accession number PRJNA1502400. Without available reference genome of common carp, de novo transcriptome assembly was performed using Trinity v2.15.1 to generate Unigenes, and clean reads were mapped back to the assembled Unigene set via RSEM v1.3.3 for read count calculation; gene expression levels were normalized to FPKM values. Differential expression analysis was implemented using the DESeq2 package v1.38.1 in R software v4.3.1, and DEGs were defined by dual thresholds of |log_2_(Fold Change)| > 1 and adjusted *p*-value (padj) < 0.05 (FDR threshold = 0.05) after multiple testing corrections to avoid relying solely on fold change, resulting in 329 DEGs including 181 upregulated and 148 downregulated genes. All Unigenes were functionally annotated against NR, GO, KEGG, eggNOG, Pfam and Swiss-Prot databases using Diamond v2.1.8, eggNOG-mapper v2.1.2 and KAAS online tool (https://www.genome.jp/kegg/kaas/, accessed on 31 July 2025), followed by GO enrichment via topGO v2.54.0 and KEGG enrichment via KOBAS v3.0 with significance set at *p* < 0.05.

### 2.9. RT-qPCR

Total RNA was extracted from cell samples following the protocol of TRIzol reagent (Vazyme Biotech Co., Ltd., Nanjing, China), and RNA concentration was determined by nucleic acid detector. Reverse transcription was performed to synthesize cDNA using HiScript II qRT Super Mix kit (Vazyme Biotech Co., Ltd., Nanjing, China). The obtained cDNA was diluted 5-fold with ddH_2_O for subsequent experiments. The *RT-qPCR* reaction system was as follows: 5 μL of 2 × Taq Pro Universal SYBR qPCR Master Mix, 0.2 μL of forward primer (10 μmol/L), 0.2 μL of reverse primer (10 μmol/L), 3.6 μL of ddH_2_O and 1 μL of cDNA template. Reaction procedures were set as initial denaturation at 95 °C for 30 s; 45 cycles of 95 °C for 10 s and 60 °C for 30 s; and melting curve stage at 95 °C for 15 s, 60 °C for 60 s and 95 °C for 15 s. β-actin was selected as the internal reference gene. Relative gene expression levels were calculated via the 2^−∆∆CT^ method.

### 2.10. Western Blot

Cell pellets were resuspended in 100 μL prechilled PBS. Appropriate volume of 5 × SDS-PAGE loading buffer was added, and samples were boiled for 12 min to extract total protein. Proteins were separated through SDS-PAGE and transferred onto PVDF membranes via the electrotransfer system. The membranes were blocked overnight at 4 °C with TBST containing 5% skimmed milk powder. Membranes were incubated with primary antibodies against *SVCV*-G, *SVCV*-N and β-actin either overnight at 4 °C or for 2 h at room temperature, followed by three 5 min washes with TBST. Subsequently, membranes were incubated with HRP-conjugated secondary antibody for 1 h at room temperature and washed three times with TBST. Protein bands were detected using ECL chemiluminescence kit and imaging system.

### 2.11. Statistical Analysis

All experiments were performed in three biological replicates with triplicate technical replicates. Data are expressed as mean ± standard deviation (SD). The Shapiro–Wilk test and Levene’s test were applied to confirm data normality and homogeneity of variances before statistical evaluation. One-way ANOVA with Tukey’s post hoc test was used for group difference analysis using GraphPad Prism 8.0. A *p*-value < 0.05 was considered statistically significant. Significance levels were defined as *, *p* < 0.05, **, *p* < 0.01, ***, *p* < 0.001.

## 3. Results

### 3.1. Effects of H. cordata Extract Concentrations on Anti-SVCV Activity in EPC Cells

**Figure 1:**
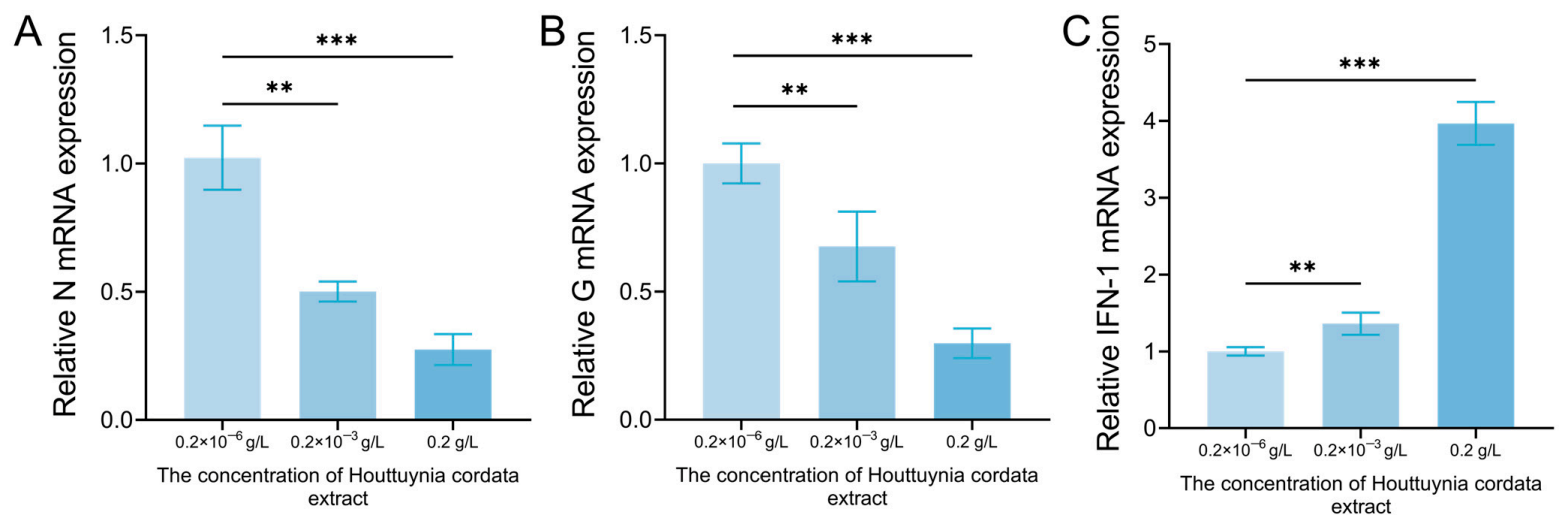
Antiviral effects of different concentrations of *H. cordata* extract on *SVCV*-infected *EPC* cells. (**A**) Relative mRNA expression levels of *SVCV*-*n* gene in *EPC* cells treated with different concentrations of *H. cordata* extract; (**B**) relative mRNA expression levels of *SVCV*-*g* gene in *EPC* cells under different *H. cordata* extract treatments; (**C**) relative mRNA expression levels of *ifn-1* gene in *EPC* cells stimulated with different concentrations of *H. cordata* extract (**, *p* < 0.01, ***, *p* < 0.001).

To verify the antiviral effect of *H. cordata* extract on *EPC* cells, three concentration gradients (0.2 g/L, 0.2 × 10^−3^ g/L, 0.2 × 10^−6^ g/L) were applied with *SVCV* infection. Cell samples were collected 24 h post-infection for RNA extraction and detection. The expression levels of *SVCV n* and *g* genes decreased along with the elevation of extract concentration (Figure 1A,B), while the expression of *IFN-1* increased. The antiviral activity exhibited a concentration-dependent manner. The concentration of 0.2 g/L was adopted for subsequent experiments.

### 3.2. Effects of H. cordata Extract Treatment Duration on Anti-SVCV Responses in EPC Cells

**Figure 2:**
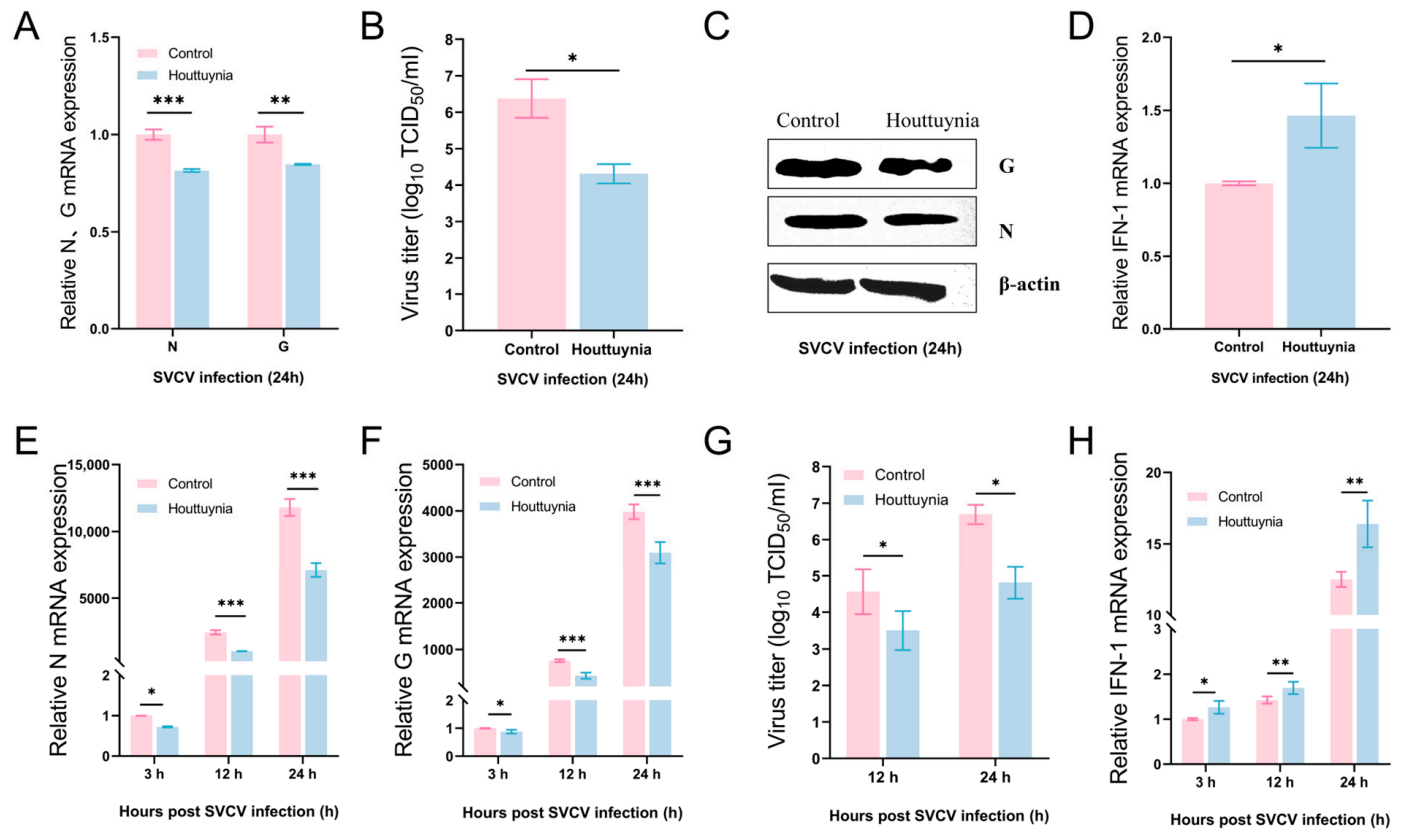
Temporal effects of *H. cordata* extract on *SVCV* infection in *EPC* cells. (**A**) Relative mRNA expression levels of *SVCV*-*n* and *SVCV*-*g* genes in *EPC* cells at 24 h post-*SVCV* infection with *H. cordata* extract treatment; (**B**) viral titers at 24 h post-SVCV infection with *H. cordata* extract treatment; (**C**) Western blot analysis of *SVCV*-N and *SVCV*-G protein expression after 24 h of *H. cordata* extract intervention and *SVCV* challenge; (**D**) relative mRNA expression level of *ifn-1* gene in *EPC* cells at 24 h post-SVCV infection with *H. cordata* extract treatment; (**E**,**F**) relative mRNA expression levels of *SVCV*-*n* and *SVCV*-g genes at 3 h, 12 h, and 24 h post-*SVCV* infection in the presence of *H. cordata* extract; (**G**) viral titers at 12 h and 24 h post-*SVCV* infection with *H. cordata* extract treatment; (**H**) relative mRNA expression level of *ifn-1* gene at 3 h, 12 h, and 24 h post-*SVCV* infection following *H. cordata* extract intervention (*, *p* < 0.05, **, *p* < 0.01, ***, *p* < 0.001).

To further verify the anti-*SVCV* ability of *H. cordata* extract in *EPC* cells, preventive administration assay and time-gradient infection assays (3 h, 12 h, and 24 h post-*SVCV* infection) were performed. The results showed that in the preventive experiment, the mRNA levels of *SVCV n* and *g* genes (Figure 2A), as well as the protein expression levels of viral structural proteins (Figure 2C), were significantly decreased in the *H. cordata* extract-treated group. Meanwhile, viral titer was markedly reduced (Figure 2B), and the expression of *ifn-1* gene was significantly upregulated (Figure 2D). In the time-gradient assay, the inhibitory effects of *H. cordata* extract on *SVCV n* and *g* gene expression were gradually enhanced with the extension of infection time. Consistently, viral proliferation was persistently suppressed, and the transcription level of *IFN-1* was significantly promoted. Collectively, these results indicated that the anti-*SVCV* effect of *H. cordata* extract exhibits a significant time-dependent manner in *EPC* cells.

### 3.3. Transcriptomic Analysis of the Inhibitory Effect of H. cordata Extract on SVCV Infection in EPC Cells

**Figure 3:**
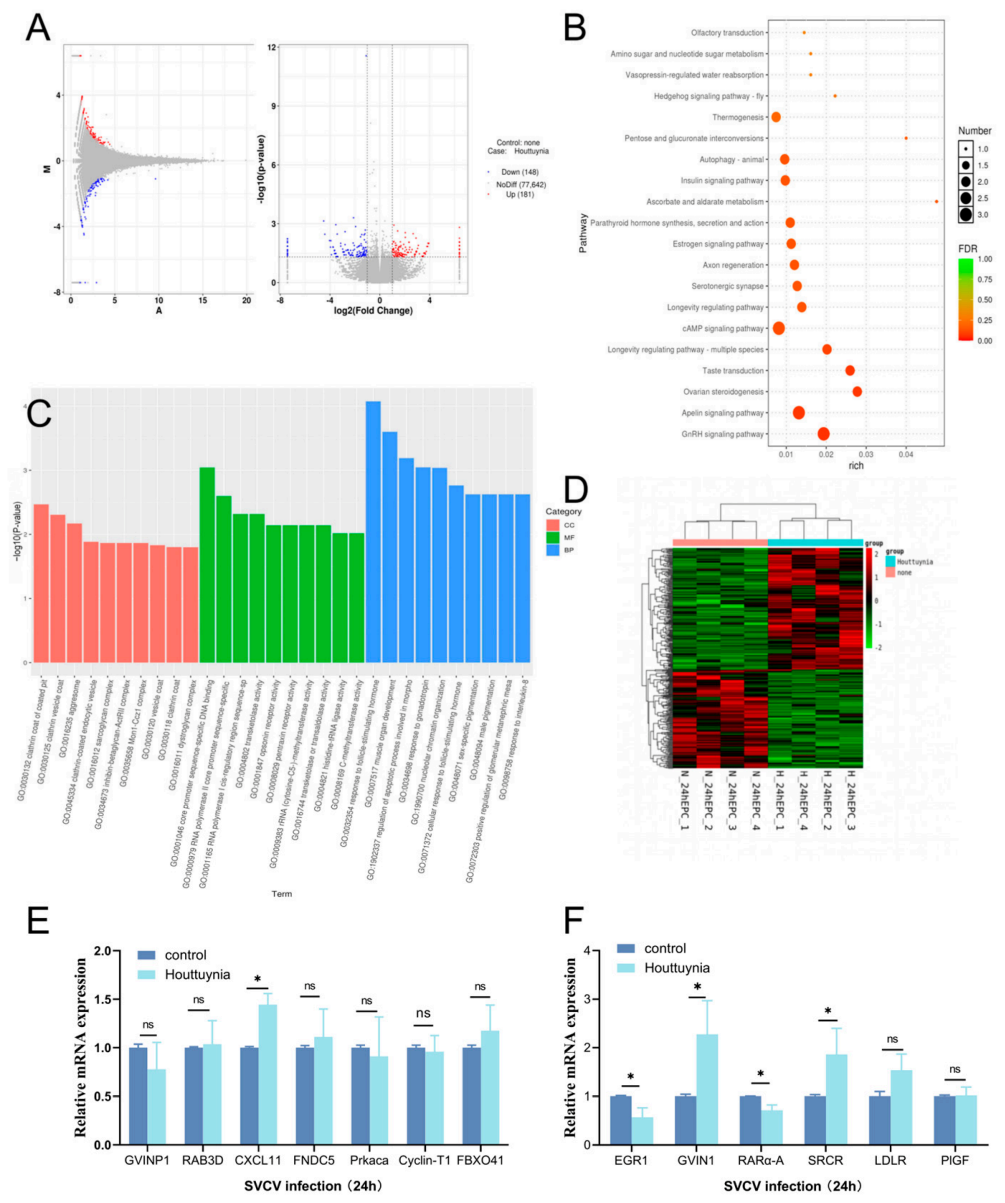
Transcriptomic profiling of *EPC* cells with or without Houttuynia treatment under *SVCV* infection at 24 h post-infection. (**A**) Volcano plot of differentially expressed genes; (**B**) KEGG enrichment bubble diagram of differentially expressed genes; (**C**) GO enrichment analysis of differentially expressed genes; (**D**) heatmap of differentially expressed genes; (**E**) relative expression levels of upregulated genes; (**F**) relative expression levels of downregulated genes (*, *p* < 0.05, ns, *p* > 0.05).

To further explore the potential molecular mechanism by which *H*. *cordata* extract exerts anti-*SVCV* effects in *EPC* cells, transcriptomic differential gene expression analysis was carried out (Figure 3A–D). Genes with a fold change (treatment group with *H. cordata* extract vs. untreated infected control group) > 2 were identified as significantly upregulated differentially expressed genes (Figure 3E), while those with a fold change < 0.5 were defined as significantly downregulated differentially expressed genes (Figure 3F) for subsequent qPCR quantitative verification. Based on the transcriptome data, two key immune-related genes were finally screened out, including the *IFN-1* upstream regulatory gene *RARα-A* and the *IFN-1* downstream effector gene *CXCL11*, which were speculated to be crucial molecules mediating the antiviral effect of *H. cordata* extract (Table A1 and Table A2).

### 3.4. Effects of RARα-A Overexpression on SVCV Infection in EPC Cells

**Figure 4:**
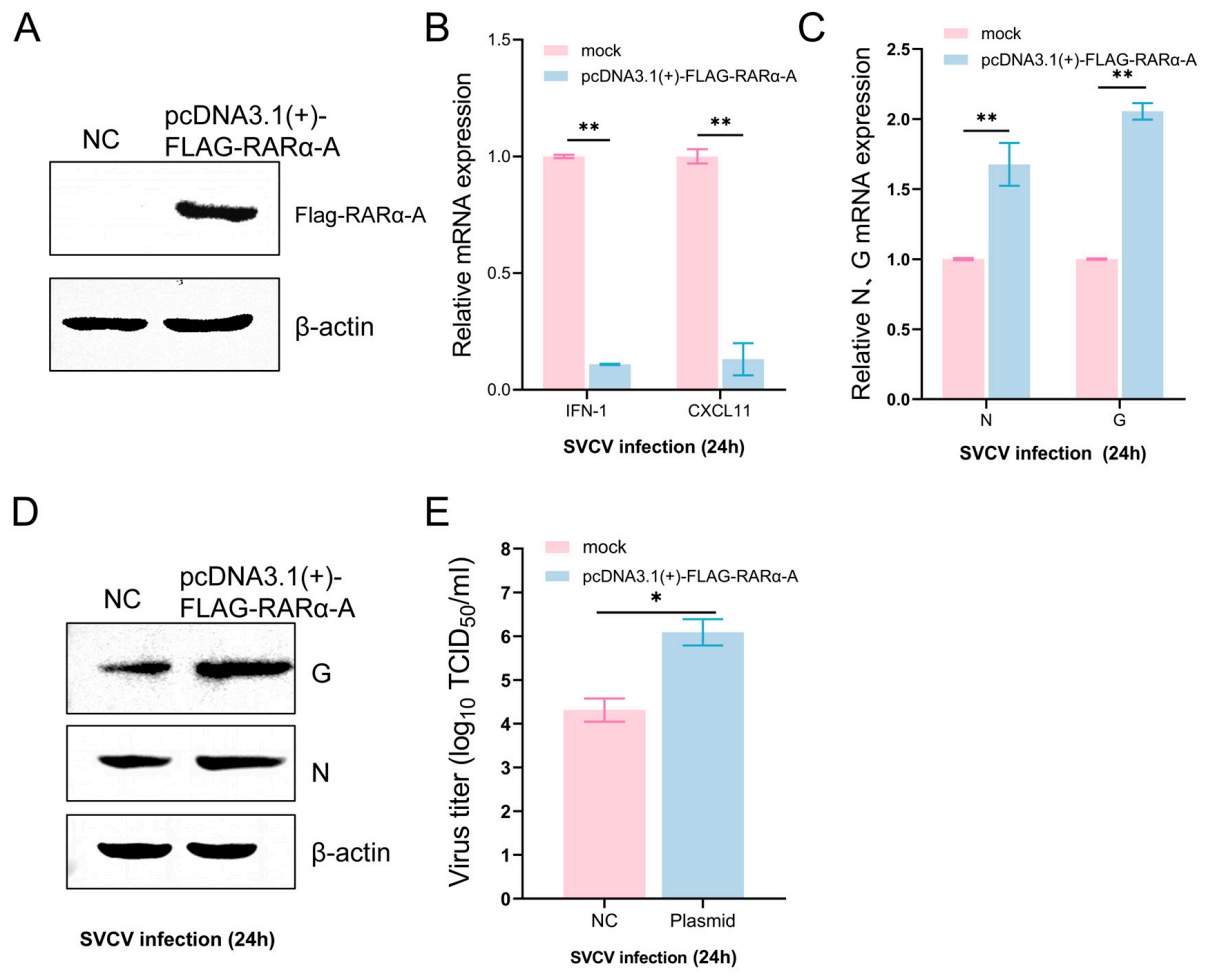
Effects of *RARα-A* overexpression on *SVCV* infection in *EPC* cells. (**A**) Verification of *RARα-A* overexpression efficiency in *EPC* cells; (**B**) relative mRNA expression levels of *IFN-1* and *CXCL11* in *EPC* cells 24 h after *SVCV* infection following *RARα-A* overexpression; (**C**) relative mRNA expression levels of *SVCV*-N and *SVCV*-G in *EPC* cells 24 h after *SVCV* infection following *RARα-A* overexpression; (**D**) protein expression levels of *SVCV*-N and *SVCV*-G in *EPC* cells 24 h after *SVCV* infection following *RARα-A* overexpression; (**E**) detection of viral titers in *EPC* cells 24 h after *SVCV* infection following *RARα-A* overexpression (*, *p* < 0.05, **, *p* < 0.01).

To investigate the regulatory role of *RARα-A* in the anti-*SVCV* response of *EPC* cells, a *RARα-A* overexpression plasmid was constructed, and an empty vector was used as the negative control. *EPC* cells were transfected with the recombinant plasmid or blank plasmid, respectively. After 24 h of cell culture, the cells were challenged with *SVCV*, and samples were collected at 24 h post-infection for *RT-qPCR* and Western blot verification. As shown in Figure 4, the transfection efficiency assay confirmed successful overexpression of *RARα-A* in *EPC* cells (Figure 4A). The results showed that the overexpression of *RARα-A* significantly downregulated the transcription levels of *IFN-1* and *CXCL11*. Meanwhile, the mRNA and protein expression of *SVCV*-N and *SVCV*-G were markedly increased, accompanied by an elevated viral titer. Collectively, these findings indicated that *RARα-A* overexpression suppresses the expression of *IFN-1* and downstream *CXCL11*, thereby weakening the antiviral capacity of *EPC* cells and facilitating *SVCV* replication.

### 3.5. Verification and Functional Analysis of RARα-A Knockdown in EPC Cells During SVCV Infection

**Figure 5:**
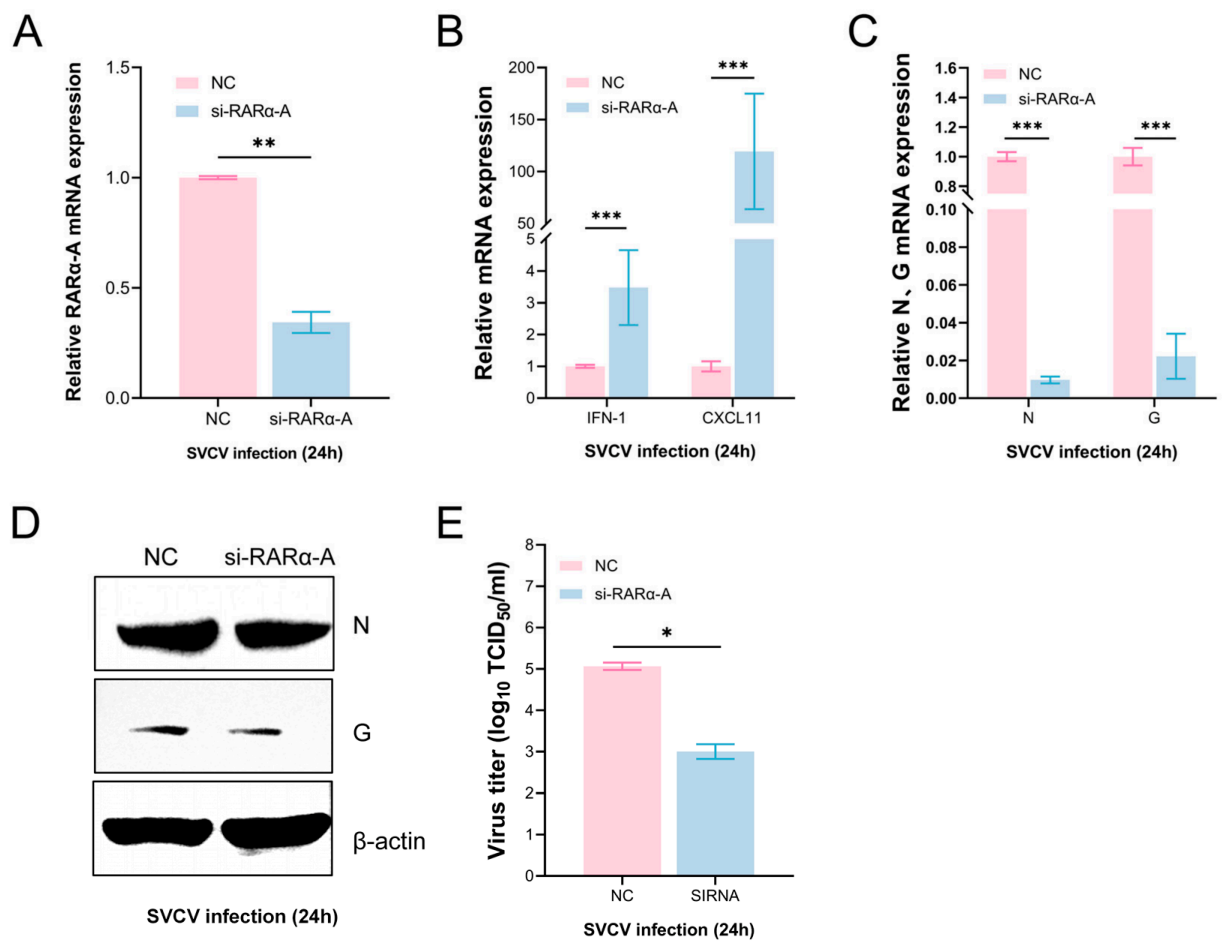
Verification and functional analysis of *RARα-A* knockdown in *EPC* cells during *SVCV* infection. (**A**) Verification of *RARα-A* knockdown efficiency; (**B**) relative mRNA expression levels of *IFN-1* and *CXCL11* after *RARα-A* silencing; (**C**) relative mRNA expression levels of *SVCV*-N and *SVCV*-G following *RARα-A* knockdown; (**D**) protein expression levels of *SVCV*-N and *SVCV*-G after *RARα-A* silencing; (**E**) detection of viral titers after *RARα-A* knockdown (*, *p* < 0.05, **, *p* < 0.01, ***, *p* < 0.001).

To further explore the regulatory function of *RARα-A* during *SVCV* infection, siRNA-mediated gene knockdown was performed to silence endogenous *RARα-A* expression in *SVCV*-infected *EPC* cells (Figure 5A). Compared with the negative control group (NC group), the mRNA levels of *IFN-1* and its downstream chemokine *CXCL11* were significantly upregulated in the *RARα-A*-knockdown group (Figure 5B), indicating that *RARα-A* negatively regulates the *IFN*-mediated antiviral immune pathway. Furthermore, the detection of viral replication levels showed that knockdown of *RARα-A* markedly inhibited the transcription and protein expression of *SVCV*-N and *SVCV*-G (Figure 5C,D) and reduced viral titers (Figure 5E). Collectively, these results demonstrate that *RARα-A* knockdown enhances host antiviral responses by promoting *IFN-1* expression, thereby effectively suppressing *SVCV* proliferation.

### 3.6. Study on the Anti-SVCV Effect of H. cordata Extract on Carp In Vivo

Given the prominent anti-*SVCV* activity of *H. cordata* extract observed in *EPC* cells, further in vivo experiments were conducted on carp. A 7-day trial revealed that *SVCV* infection mainly induced hemorrhagic lesions in gill and muscle tissues of carp. The immune factors of the two tissues were subsequently analyzed to evaluate the antiviral efficacy of the extract against *SVCV*.

**Figure 6:**
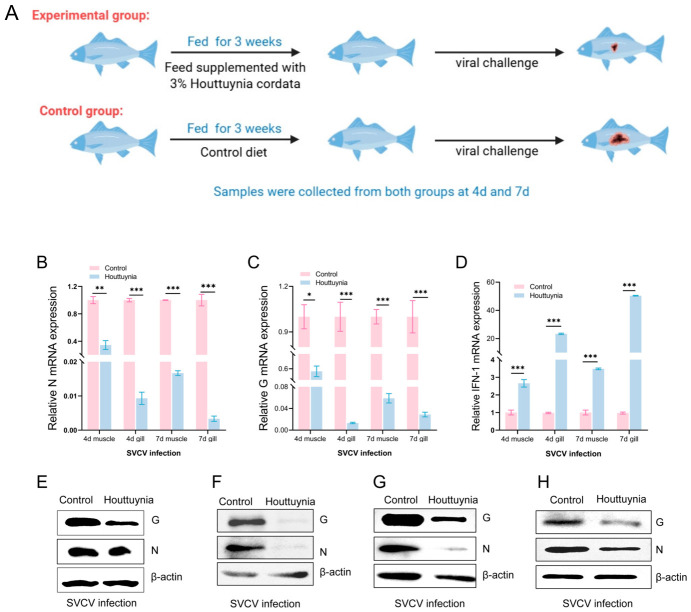
Results of in vivo experiments in carp. (**A**) Flowchart of in vivo rearing experiment; (**B**) relative expression of *n* gene in carp muscle and gill tissues; (**C**) relative expression of *g* gene in carp muscle and gill tissues; (**D**) relative expression of *ifn-1* gene in carp muscle and gill tissues; (**E**) Western blot of N and G proteins in carp muscle at 4 days post-infection; (**F**) Western blot of N and G proteins in carp gill at 4 days post-infection; (**G**) Western blot of N and G proteins in carp muscle at 7 days post-infection; (**H**) Western blot of N and G proteins in carp gill at 7 days post-infection (*, *p* < 0.05, **, *p* < 0.01, ***, *p* < 0.001).

Juvenile carp weighing 55 ± 5 g were used for in vivo verification, and the experimental procedure is presented in Figure 6A. Preliminary tests showed that obvious hemorrhagic lesions occurred in gills and somatic muscles at 4 days post-infection, and fish mortality emerged at 7 days post-infection. Accordingly, muscle and gill samples were collected at 4 d and 7 d post challenge for quantitative and protein expression detection. The results demonstrated that both mRNA and protein levels of *SVCV*-N and *SVCV*-G were reduced in the two tissues. Meanwhile, the expression of *IFN-1* was significantly upregulated in carp, and the increasing tendency became more pronounced over time. These data demonstrate that *H. cordata* extract elevates *IFN-1* expression and inhibits *SVCV* replication in carp.

## 4. Discussion

In previous studies, retinoic acid receptor alpha-A (*RARα-A*), an upstream regulator of *IFN-1*, has been demonstrated to inhibit *IFN-1* expression [12]. C-X-C motif chemokine ligand 11 (*CXCL11*) acts as a downstream factor of *IFN* [13]. Its expression can be markedly upregulated under interferon induction, and it participates in antiviral immunity by regulating chemotaxis and polarization of immune cells [14]. Several studies have further confirmed that *CXCL11* plays a vital role in antiviral immune responses [15,16,17]. This study was conducted via *EPC* cell assays and carp in vivo experiments. The results imply that *H. cordata* extract may upregulate the expression of *IFN-1* and its downstream chemokine *CXCL11*, potentially through the *RARα-A* signaling cascade, and exert effective suppressive effects on the gene transcription, protein synthesis and replication of *SVCV* both in vitro and in vivo. Consistent with the general pattern of herbal antiviral immunity, multiple studies have indicated that natural herbal extracts suppress *SVCV* mainly via modulating interferon-centered innate immune pathways rather than directly eliminating virions [18,19,20,21]. Previous work also reports that *H. cordata* can trigger the mTORC1 pathway through p38 MAPK phosphorylation, thereby enhancing *IFN-1* signaling activation [22]. Collectively, the above studies indicate that natural Chinese herbal medicines exert anti-*SVCV* effects mainly by targeting *IFN*-centered immune signaling pathways to activate host immune responses and synergistically inhibit viral replication. These findings provide sufficient theoretical support and comparative evidence for the antiviral mechanism of *H. cordata* extract demonstrated in the present study.

**Figure 7:**
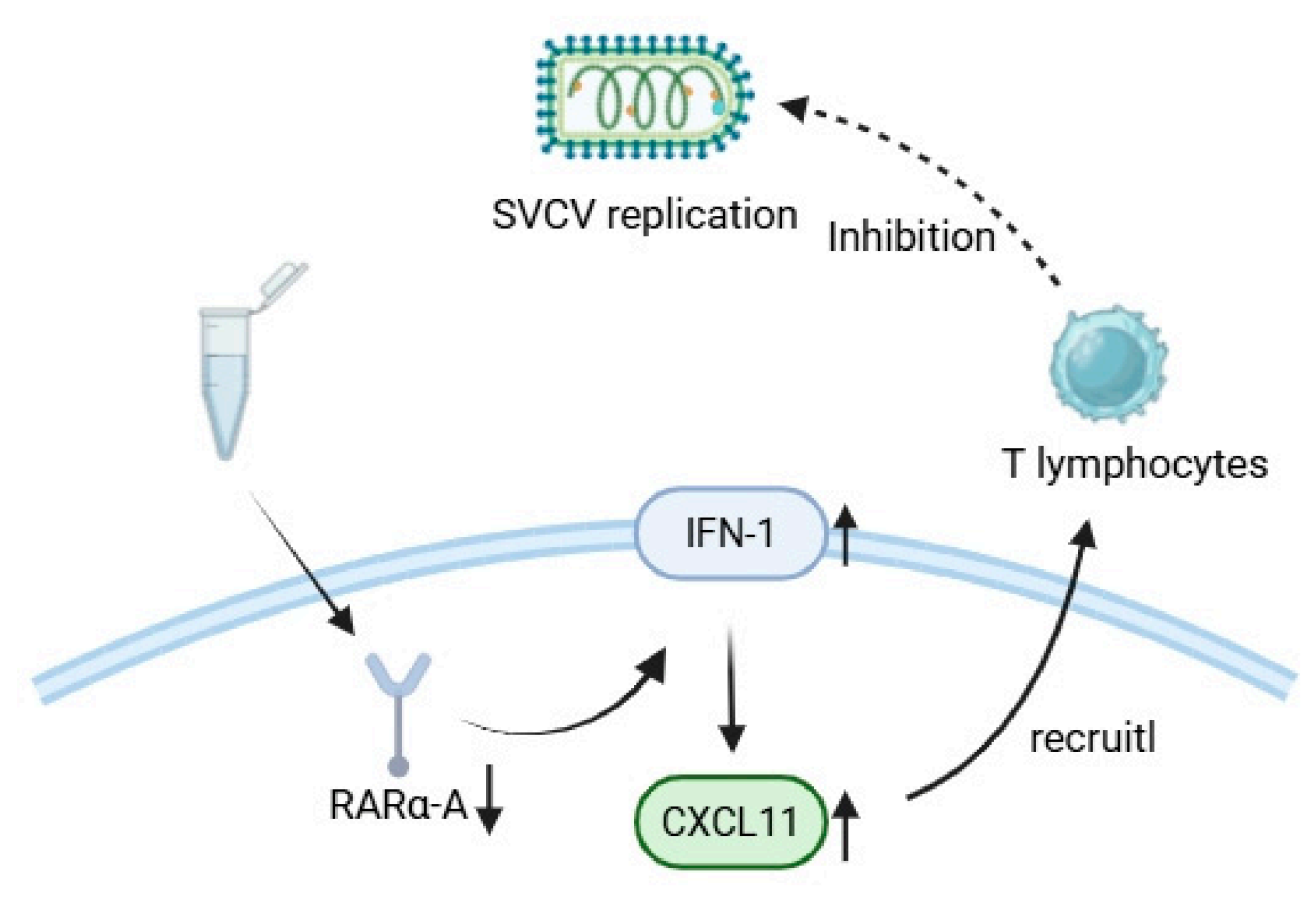
Schematic diagram of the antiviral mechanism of *H. cordata* extract against *SVCV*.

The biological activities of *H. cordata* are attributed to its complex chemical composition [23]. Its major active constituents have been confirmed to include volatile oils, flavonoids, polysaccharides, and polyphenols [24]. These diverse components exert immunomodulatory, antibacterial, and antiviral functions through synergistic interactions [25]. Most prior studies concerning *H. cordata* have primarily focused on its influences on routine immune indices and growth performance of fish [26], while several studies have merely validated its regulatory effects on the antioxidant capacity of cyprinid fish [27]. In terms of action mechanism, existing studies have indicated that the medicinal plant heteropolysaccharide HCPM from *H. cordata* can enrich intestinal *Phocaeicola vulgatus* and promote acetic acid production, thereby alleviating acute lung injury induced by H1N1 virus in mice [28]. *H. cordata* can inhibit dexamethasone-induced apoptosis and inflammatory responses of bone marrow mesenchymal stem cells in vitro and downregulate the levels of key proteins in the *NF-κB* pathway. In vivo, it alleviates pathological damage and reduces the expression of apoptotic and inflammatory factors in rats with steroid-induced avascular necrosis of the femoral head [29]. *H. cordata* extract can effectively alleviate pathological damage, protect intestinal barrier, reduce bacterial load and regulate inflammation in mice and chicken models infected with Salmonella. It exerts effects through downregulating HilA, a key regulator of T3SS 1, and inhibiting effector protein secretion and bacterial invasion [9]. In contrast, the present study based on in vitro cell models reveals that *H. cordata* extract exerts anti-*SVCV* activity, and the *RARα-IFN* signaling pathway is potentially involved in this antiviral process, which partially fills the research gap regarding the molecular mechanism underlying the anti-*SVCV* function of *H. cordata* extract (Figure 7). Moreover, this study validates the specific target of *H. cordata* extract. Previous studies have mostly attributed the immunomodulatory capacity of *H. cordata* to its broad-spectrum anti-inflammatory and antibacterial activities [30], while failing to further clarify its association with key immune transcription factors in fish. Notably, this study preliminarily identifies *RARα-A* as a potentially involved regulatory factor that participates in mediating the antiviral activity of *H. cordata* extract, which provides a novel theoretical basis for the targeted screening and precise development of natural plant-derived immunostimulants.

To investigate the immunomodulatory mechanism of *H. cordata* extract in common carp, the present study demonstrated that it exerts antiviral immune functions by modulating the potential *RARα*-*IFN* signaling pathway. Previous studies have shown that all-trans retinoic acid, 9-cis retinoic acid, and the clinical ligand 13-cis retinoic acid promote the expression of CD69 and CD38 in activated human T cells via retinoic acid receptor α, upregulate type 2 cytokines, and simultaneously inhibit *IFN*-γ expression [31]. Building on this, the present study, through *RARα-A* knockdown and overexpression experiments, established a complete regulatory pathway in which *H. cordata* extract employs *RARα-A* as a key regulatory node, subsequently modulating interferon signaling and suppressing *SVCV* replication. These findings identify *RARα-A* as a critical intermediary target mediating the antiviral effects of *H. cordata* extract, elucidate the molecular mechanism by which natural plant products regulate fish immunity, and provide a precise target for the future development of targeted antiviral agents. Moreover, this study confirmed that *H. cordata* extract simultaneously suppresses viral gene transcription and protein synthesis, thereby offering a more comprehensive assessment of its actual anti-*SVCV* efficacy.

Nevertheless, certain limitations of the current study need to be objectively addressed. We only characterized the mRNA-level transcriptional alterations of *RARα-A* and *CXCL11* through transcriptome sequencing and *RT-qPCR*. It should be emphasized that the biological function of *CXCL11* in the RARα-A/*IFN-1*/*CXCL11* axis is only hypothesized based on transcriptional data and published references and lacks direct experimental verification. Functional cellular and molecular experiments such as gene knockout were not performed to clarify their direct regulatory functions and molecular interaction mechanisms in the *RARα-A*/*IFN-1*/*CXCL11* antiviral signaling cascade. In follow-up work, our research group will carry out in vitro and in vivo functional verification assays targeting *RARα-A* and *CXCL11*. Specifically, *CXCL11* knockout and overexpression cell lines, lymphocyte chemotaxis assays, and gene-edited animal models will be constructed to verify its immune recruitment and indirect anti-*SVCV* effects, so as to further dissect the underlying regulatory mechanism by which these two molecules mediate the protective efficacy of *H. cordata* extract against *SVCV* infection in common carp.

With increasingly stringent supervision on aquatic product quality and safety and growing public health awareness, green, eco-friendly and residue-free aquaculture has become the core direction for sustainable development of carp industry [32]. This provides broad market prospects for the application of *H. cordata* as a natural feed additive. In traditional aquaculture models, the long-term application of chemical drugs easily causes a series of problems, including drug residues, pathogenic bacterial resistance, and aquatic microecological imbalance, which severely restrict industrial development and market competitiveness of aquatic products. Compared with chemical agents, *H. cordata*, as a medicinal and edible homologous plant [33], can fundamentally prevent the risk of drug residues, provide safe and healthy carp products for consumers, and conform to the development trend of the organic food market. The findings of this study preliminarily reveal the immune-regulatory and anti-*SVCV* effects of *H. cordata* extract in common carp. This extract serves as a promising candidate that deserves further systematic evaluation as a natural immunomodulatory feed additive for green aquaculture.

## 5. Conclusions

**Figure A1:**
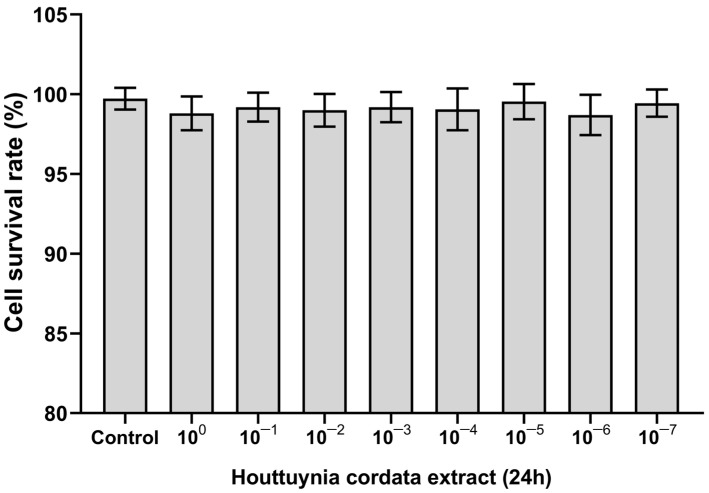
Cell viability of *EPC* cells after 24 h treatment with *H. cordata* extract. *EPC* cells were cultured in complete M199 medium supplemented with 10% fetal bovine serum (FBS) at 26 °C. The cells were seeded in 96-well plates at a density of 1 × 10^4^ cells per well and incubated overnight for full adherence. *H. cordata* extract was serially diluted with culture medium (ranging from 10^0^ to 10^−7^), and the diluted extract was added at a volume ratio of 1:1000. A blank solvent group was set as the control, with six biological replicate wells for each concentration. After 24 h of incubation with the extract, 10 μL of CCK-8 reagent (Beyotime Biotechnology Co., Ltd., Shanghai, China) was added to each well, followed by a further 2 h incubation at 26 °C. The absorbance was measured at 450 nm using SpectraMax ABS Plus absorbance microplate reader (Molecular Devices, San Jose, CA, USA) to calculate cell viability: cell viability (%) = (OD value of the treatment group/OD value of the control group) × 100%. The viability of *EPC* cells remained above 95% after 24 h of treatment with *H. cordata* extract, indicating no obvious cytotoxicity of the extract against *EPC* cells.

**Figure A2:**
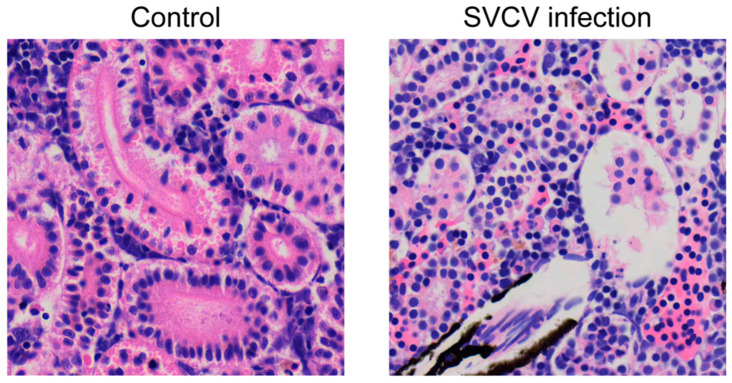
Histopathological observation of carp trunk kidney tissue after *SVCV* infection.

*H. cordata* extract effectively blocks *SVCV* replication in *EPC* cells, and all extract concentrations adopted in this antiviral assay exhibit negligible cytotoxicity to *EPC* cells after 24 h treatment (Figure A1). In vivo feeding trials confirm that this extract improves the immunoregulatory ability of common carp and lowers viral loads in fish tissues. Mechanistic analysis revealed that *H. cordata* extract promotes the activation of type I interferon signaling cascades by downregulating the potential factor *RARα-A*, which in turn upregulates the downstream immune mediator *CXCL11*. Subsequently, *CXCL11* recruits T lymphocytes to initiate adaptive antiviral immunity [34], collectively suppressing the replication and proliferation of the rhabdovirus *SVCV*. The present study preliminarily indicates that *RARα-A* may serve as a potential regulatory molecule linked to the anti-*SVCV* effect of *H. cordata* extract. These results could offer a tentative theoretical reference, and further comprehensive assessments are still required before *H. cordata* can be developed as a natural feed additive to alleviate *SVCV* infection in cyprinid farming.

## Figures and Tables

**Table 1:** Primer sequences.

Primer Name	Sequences (5′ → 3′)
β-actin-FW	5′-CCGTGACCTGACTGAATACC-3′
β-actin-BW	5′-GCCCATCTCCTGCTCAAAG-3′
SVCV-G-FW	5′-CGACCTGGATTAGACTTG-3′
SVCV-G-BW	5′-AATGTTCCGTTTCTCACT-3′
SVCV-N-FW	5′-AACAGCG CGTCTTACATGC-3′
SVCV-N-BW	5′-CTAAGGCGTAAGCCATCAGC-3′
IFN-1-FW	5′-ACCAAACCCAAATGTGGACGTG-3′
IFN-1-BW	5′-CCACTCATTTCCCGAAGCAGA-3′
EPC-IFN-1-FW	5′-ATGTATCGCTTTGCTGTGTC-3′
EPC-IFN-1-BW	5′-TTCGGGAAGGTTATTTTAAC-3′
CXCL11-FW	5′-CGGATGTTCATAAGCAAG-3′
CXCL11-BW	5′-TTTAGGATTCAGGCAGAC-3′
RARα-A-FW	5′-CCCTCACCATCGCAGACCA-3′
RARα-A-BW	5′-CGGACCGAAGCCAGCATT-3′

## Data Availability

The raw experimental data generated in this study are available from the corresponding author upon reasonable request.

## Appendix A

**Table A1:** DEG screening.

	ID	Genes Name
foldchange (Houttuynia/none) < 0.5	TRINITY_DN356_c6_g1	Early growth response protein 1
	TRINITY_DN3262_c4_g1	Interferon-induced very large GTPase1-like isoform X1
	TRINITY_DN10360_c2_g1	Retinoic acid receptor alpha-A
	TRINITY_DN18550_c0_g1	Scavenger receptor cysteine-rich type 1 protein M130-like
	TRINITY_DN40849_c0_g1	Low-density lipoprotein receptor-related protein
	TRINITY_DN42616_c0_g1	Placenta growth factor
foldchange (Houttuynia/none) > 2	TRINITY_DN8918_c0_g2	Interferon-induced very large GTPase 1-like
	TRINITY_DN9356_c0_g1	RAB3D, member RAS oncogene family, b isoform X1
	TRINITY_DN19509_c0_g1	C-X-C motif chemokine 11
	TRINITY_DN34874_c0_g2	Fibronectin type III domain-containing protein
	TRINITY_DN40345_c0_g1	Protein kinase, cAMP-dependent, catalytic, beta a
	TRINITY_DN49896_c0_g1	Cyclin-T1
	TRINITY_DN54335_c0_g2	F-box only protein 41-like
	TRINITY_DN8918_c0_g2	Interferon-induced very large GTPase 1-like

**Table A2:** Primer sequences.

Gene Names	Sequences (5′→3′)
EPC-EGR1	AGCGACCATCTGACGACC
	CCTTCTCCGCCTTCTTGT
EPC-RARAA	CCCTCACCATCGCAGACCA
	CGGACCGAAGCCAGCATT
EPC-CIPC_	CGACGCTGTTATGATGAA
	GGTGAAGTGGTGGTATGG
EPC-GVIN1	AATCACAGACATCGCTAA
	TTCCCTCTTGAAGACATA
EPC-RAB3D	GGGCTTCCTGCTAATGTA
	CGTTATCCCACGAGTATGT
EPC-FNDC7	ATGTGACCGCAGTGATGG
	TGTGAACAGTCCCGAACC
EPC-KAPCA	TCAGCAAGGGCTACAACA
	CAGCGAAGAAAGGTGGAT
EPC-GVIN1	GTGCCACATCTACCTTTA
	TCTGAAATCGCTGACTGA
EPC-M130-like	GCGACTGTGACCACCAAC
	GCGTCCAGCAGAAGATGA
EPC-L-DLRRP	TGCGACAGCAGTCCAGGTT
	TGGCTCCAGAATGGGTTT
EPC-C-X-C	CGGATGTTCATAAGCAAG
	TTTAGGATTCAGGCAGAC
EPC-Cyclin-T1	TTGAGTACAGTAGGGATGT
	ATGCCAGTAGTTGAAACC
EPC-FBX41	GAGCCGTTATGTTTGGGTG
	GAGGAGCGATCCGAAGTG
